# Does hybridization with a widespread congener threaten the long‐term persistence of the Eastern Alpine rare local endemic *Knautia carinthiaca*?

**DOI:** 10.1002/ece3.1686

**Published:** 2015-09-09

**Authors:** Martin Čertner, Filip Kolář, Peter Schönswetter, Božo Frajman

**Affiliations:** ^1^ Department of BotanyFaculty of ScienceCharles University in PragueBenátská 2CZ‐128 00PragueCzech Republic; ^2^ Institute of BotanyThe Czech Academy of SciencesZámek 1CZ‐252 43PrůhoniceCzech Republic; ^3^ National Centre for BiosystematicsNatural History MuseumUniversity of OsloNO‐0318OsloNorway; ^4^ Institute of BotanyUniversity of InnsbruckSternwartestraße 156020InnsbruckAustria

**Keywords:** Eastern Alps, habitat segregation, human‐induced landscape changes, introgression, relict, steno‐endemic species

## Abstract

Interspecific hybridization, especially when regularly followed by backcrossing (i.e., introgressive hybridization), conveys a substantial risk for many endangered organisms. This is particularly true for narrow endemics occurring within distributional ranges of widespread congeners. An excellent example is provided by the plant genus *Knautia* (Caprifoliaceae): Locally endemic *K*. *carinthiaca* is reported from two isolated populations in southern Austria situated within an area predominantly occupied by widespread *K*. *arvensis*. While *K*. *carinthiaca* usually inhabits low‐competition communities on rocky outcrops, *K*. *arvensis* occurs mainly in dry to mesic managed grasslands, yet both species can coexist in marginal environments and were suspected to hybridize. Flow cytometry revealed that diploid *K*. *carinthiaca* only occurs at its locus classicus, whereas the second locality is inhabited by the morphologically similar but tetraploid *K*. *norica*. In the, therefore, single population of *K*. *carinthiaca,* flow cytometry and AFLP fingerprinting showed signs of introgressive hybridization with diploid *K*. *arvensis*. Hybridization patterns were also reflected in intermediate habitat preferences and morphology of the hybrids. Environmental barriers to gene flow seem to prevent genetic erosion of *K*. *carinthiaca* individuals from the core ecological niches, restricting most introgressed individuals to peripheral habitats. Efficient conservation of *K. carinthiaca* will require strict protection of its habitat and ban on forest clear cuts in a buffer zone to prevent invasion of *K. arvensis*. We demonstrate the large potential of multidisciplinary approaches combining molecular, cytometric, and ecological tools for a reliable inventory and threat assessment of rare species.

## Introduction

Although hybridization is widely accepted as a common and important evolutionary force in plants (Stebbins 1950; Lewontin and Birch 1966; Rieseberg et al. 2003; Soltis and Soltis 2009; Ellstrand 2014; Yakimowski and Rieseberg 2014), its impact on biodiversity is varied. On the one hand, hybridization may lead to phenotypic novelty and result in the formation of new species (Rieseberg 1997; Givnish 2010; Renaut et al. 2014). Alternatively, the same process may cause a breakdown of species integrity and ultimately drive species to extinction (Rieseberg et al. 1989; Levin et al. 1996; Rhymer and Simberloff 1996; Rieseberg and Carney 1998; Rosenfield et al. 2004). Hybridization is perhaps the most rapidly acting genetic threat to endangered species, with extinction predicted to take place in theoretically less than five generations (Wolf et al. 2001). Hence, hybridization is being referred to as the “double‐edged sword of conservation biology” (Haig and Allendorf 2006). Finally, interspecific mating can also result in locally and temporarily restricted hybrid zones without significantly affecting the integrity of the involved species (Mayr 1992).

Whereas in some plant groups interspecific hybridization leads to nonreproducing first‐generation hybrids only (Jackson et al. 1993; Vít et al. 2014), hybrids backcross with parental species in other groups. This so‐called introgressive hybridization (Anderson 1949; Futuyma 1998) may ultimately lead to genetic erosion of the parental gene pools (Wolf et al. 2001). Human influence has strongly fostered interspecific hybridization as alteration of natural ecosystems such as deforestation followed by spread of grassland species usually leads to invasion of locally alien species and promotes their hybridization with native congeners (e.g., Brochmann 1984; Lehman et al. 1991; Vilà et al. 2000; Levin 2002; Mallet 2005; Keller et al. 2008; Vít et al. 2014). While introgressive hybridization likely has only local impact on widely distributed species, it may pose a serious threat for local endemics and small isolated populations (Avise and Hamrick 2001; Thompson et al. 2010), where frequent pollen swamping from widespread congeners eventually results in extinction of unique evolutionary entities (Rieseberg and Gerber 1995).

Floristic surveys indicate that ca. 10% of plant species hybridize (Yakimowski and Rieseberg 2014), but the incidence of hybridization is unevenly distributed across taxonomic groups, with hybrids reported in approximately 40% of families and 16% of genera (Ellstrand et al. 1996; Whitney et al. 2010). A genus notorious for the high incidence of hybridization is *Knautia* (Caprifoliaceae; Szabó 1911; Hayek 1914; Ehrendorfer 1962; Štěpánek 1997; Kolář et al. 2012; Rešetnik et al. 2014), which includes a few widespread species, but most of the taxa are confined to small areas (Ehrendorfer 1962, 1976; Rešetnik et al. 2014). The species with the widest distribution, ranging almost throughout the entire range of the genus, is *K. arvensis* (L.) Coult. This species occurs in a variety of different habitats spanning from meadows and pastures to ruderal sites and forest clearings (Ehrendorfer 1976; Fischer et al. 2008; authors' personal observations) and most likely expanded its range dramatically during the last few thousand years in the course of the expansion of secondary grasslands following anthropogenic deforestation (Rešetnik et al. 2014). On the other hand, one of the geographically most restricted *Knautia* species is *K. carinthiaca* Ehrend., which is endemic to the valleys of the streams Görtschitz and Gurk in the Austrian federal state of Carinthia (Kärnten; Fischer et al. 2008). Only two isolated populations in the vicinity of the villages Eberstein and Launsdorf are known, separated by ca. 8 km (Ehrendorfer 1962; Schratt‐Ehrendorfer 2009).

**Figure 1:**
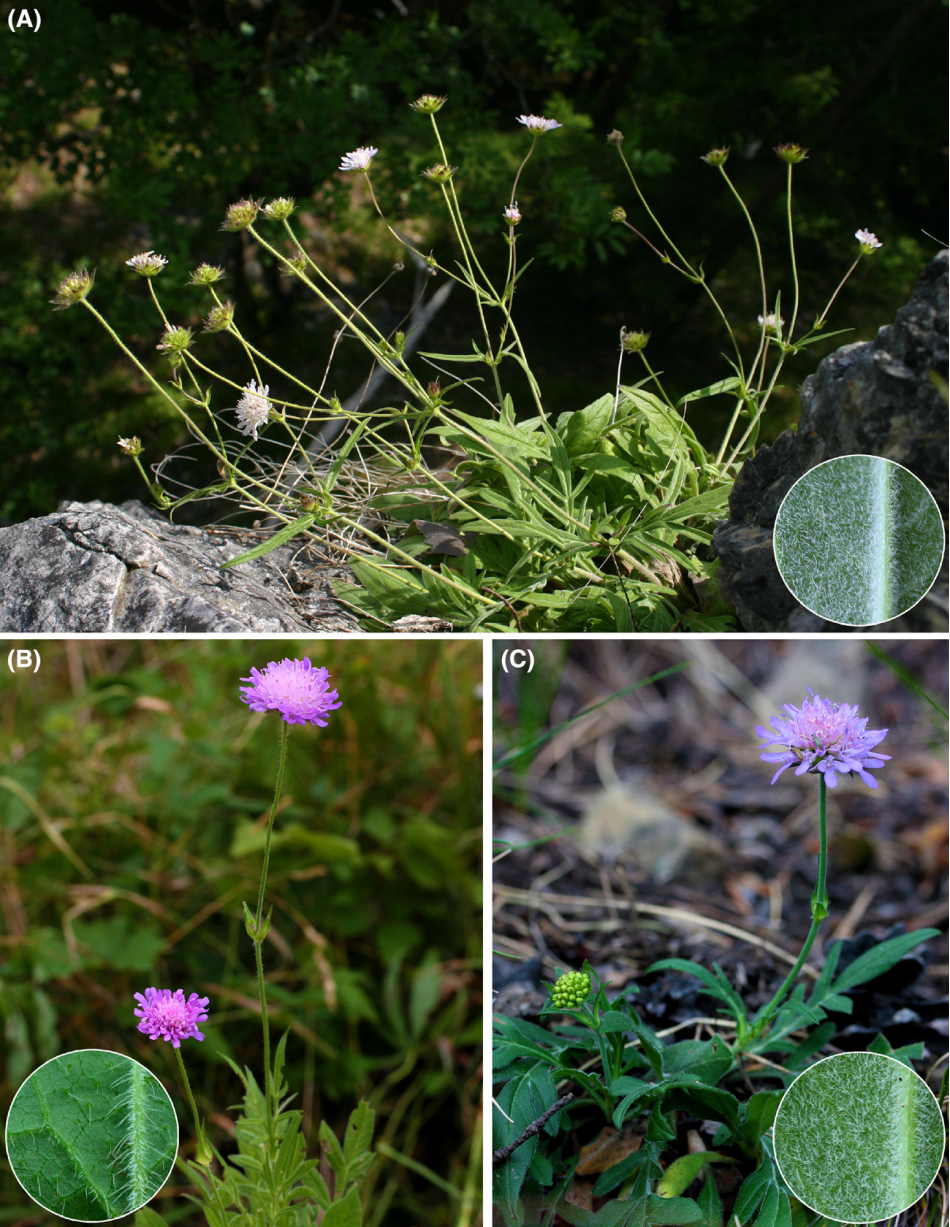
The two studied diploid species, locally endemic *Knautia carinthiaca* (A) and widespread *K*. *arvensis* (B). The former is one of the presumed parents of allotetraploid *K*. *norica* (C); that species and *K*. *carinthiaca* were reported to coexist on Mt. Otwinskogel. Circular insets show the hair density on the abaxial leaf surface close to the mid rib.

*Knautia carinthiaca* was only relatively recently recognized as an independent, locally endemic diploid (2*n* = 2*x* = 20) species (Ehrendorfer 1962). This was confirmed by molecular data suggesting that *K. carinthiaca* has an isolated position among the diploid taxa of the genus (Rešetnik et al. 2014). It inhabits mosaic habitats comprising calcareous cliffs and screes as well as adjacent dry grasslands and open thermophilous forests. However, *K. carinthiaca* also occurs in extensively mown and/or grazed semi‐dry grasslands at the periphery of its core habitats, where it coexists with diploid *K*. *arvensis*. Based on field observations, both species were suggested to hybridize (Fischer et al. 2008; Schratt‐Ehrendorfer 2009). Morphologically, *K. carinthiaca* differs from *K. arvensis* in its dense, soft indumentum giving a whitish appearance to the leaves, a lower number of lateral lobes of the stem leaves, and a terminal leaf segment, which is as long as the divided part of the leaf (Fig. 1; Fischer et al. 2008). In addition to *K. arvensis*, the range of *K. carinthiaca* overlaps with tetraploid (2*n* = 4*x* = 40) *K*. *drymeia* Heuff. and *K*. *norica* Ehrend. The latter is morphologically similar to *K. carinthiaca* (Fig. 1) as it is a putative allotetraploid between *K. carinthiaca* and *K*. *drymeia* (Ehrendorfer 1962; Fischer et al. 2008). Based on field observations, Melzer (1973) suggested that on Mt. Otwinskogel near Launsdorf *K. carinthiaca* gradually intergrades with *K. norica* toward the east and the west. Across‐ploidy mating of *K. carinthiaca* with *K. drymeia* and *K. norica* is, however, improbable due to the strong triploid block typical for *Knautia* (Ehrendorfer 1962; Breton‐Sintès 1974).

The first aim of our study was to improve the knowledge of distribution and ecology of *K. carinthiaca* and investigate its presence on Mt. Otwinskogel near Launsdorf, from where both *K. carinthiaca* and *K. norica* have been reported. After delimiting the distribution area of *K. carinthiaca*, our main aim was to assess the frequency of hybridization with *K. arvensis*, which is an important prerequisite for an efficient conservation planning. Based on field surveys, vegetation assessments, relative genome size (RGS) measurements, amplified fragment length polymorphism (AFLP) fingerprinting, and scoring of morphological characters, we addressed the following questions: (1) Are geographically adjacent populations of *K. carinthiaca* and *K. arvensis* genetically distinct and if so, what is the exact geographic distribution of *K. carinthiaca*? (2) Is there genomic (AFLP) and/or cytological (RGS) evidence for (introgressive) hybridization between *K. arvensis* and *K. carinthiaca*? (3) If so, are hybrids restricted to intermediate habitats at the periphery of *K. carinthiaca* populations or is there evidence for the occurrence of hybrids in core habitats? (4) Does morphological variability reflect possible interspecific hybridization and could thus serve as a reliable marker for hybridisation risk assessment? and (5) Finally, we evaluate if the long‐term persistence of *K. carinthiaca* is threatened and propose necessary conservation actions.

## Material and Methods

### Field survey and collection of plant material

The only two known localities of *K. carinthiaca*, Mt. Gutschenkogel W of Eberstein (46°48′14″N, 14°33′01″E; type locality) and Mt. Otwinskogel N of Launsdorf (46°46′34″N, 14°27′24″E; Ehrendorfer 1962; Schratt‐Ehrendorfer 2009) in Kärnten, southern Austria, were visited in June 2013. We intended to sample individuals from all occupied habitats. Sampling from rock crevices was partly performed on belay; however, large vertical rock faces were omitted from our study. As on Mt. Otwinskogel no *K. carinthiaca* was found among 50 individuals analyzed by flow cytometry (see Results), this locality is disregarded in the following. In addition to *K. carinthiaca*, we included adjacent populations of *K. arvensis*. In total, 118 individuals were sampled at Mt. Gutschenkogel, including *K*.* carinthiaca*,* K*. *arvensis,* and plants of intermediate morphology; this locality is further divided into two sub‐localities separated by ca. 900 meters, which are termed NE locality (46°48′14″N, 14°33′07″E) and SW locality (46°47′53″N, 14°32′31″E) in the following. For each sampled individual, we recorded accompanying vascular plant species (except other *K. carinthiaca* and *K. arvensis* individuals) rooting in a 0.3‐m radius across the target individual, assessed relative coverage of moss layer, herb layer, and bare rock, and collected leaf tissue for DNA extraction and RGS measurements. When possible, we collected one fully developed lower stem leaf per plant for morphological comparison. Documentation of flower color was not possible as only a few plants were flowering during our field campaign. For the evaluation of interspecific hybridization, our sampling for genetic analyses was supplemented by nine individuals from two more distant *K*. *arvensis* populations (W of Fiming [46°45′48.0″N, 14°25′05.6″E], NW of St. Florian [46°48′12.9″N, 14°30′40.5″E] at 10 and 2.5 km distance from Mt. Gutschenkogel). Voucher specimens are stored in the Herbarium of the University of Innsbruck (IB).

### Estimation of relative genome size

Relative genome size (RGS) was inferred from fluorescence intensities of DAPI‐stained nuclei using flow cytometry, following Hanzl et al. (2014). We chose *Bellis perennis* L. as internal reference standard, as its genome size (2C = 3.38 pg DNA; Schönswetter et al. 2007) is close to that of the studied species. Fresh leaf tissue of sample and internal standard was processed together in a two‐step procedure using Otto I and II buffers (Otto 1990), and the extracted nuclei were stained with DAPI. Fluorescence intensity of at least 3,000 particles was assessed using a Partec PA II flow cytometer (Partec GmbH, Münster, Germany) equipped with a UV LED chip as source of UV excitation light. Samples were re‐analyzed if the quality of resulting histograms was insufficient (i.e., coefficient of variation of G_0_/G_1_ peak of *Knautia* sample >3%). Due to low variability in RGS among samples from the SW locality of *K*. *carinthiaca*, 16 randomly chosen individuals (47%) were omitted from the RGS analyses. The reliability of flow‐cytometric measurements (i.e., between‐plant differences) was repeatedly confirmed in simultaneous runs of *K. arvensis* and *K. carinthiaca* accessions.

### DNA extraction and AFLP fingerprinting

Total genomic DNA was extracted from equal amounts (ca 10 mg) of silica gel‐dried leaf tissue using the DNeasy 96 Plant Mini Kit (Qiagen, Hilden, Germany) following the manufacturer's instructions. The AFLP protocol followed Vos et al. (1995) with the modifications described in Schönswetter et al. (2009) and Rešetnik et al. (2014). We employed the same three primer combinations for the selective PCR as in the previous study (Rešetnik et al. 2014; fluorescent dye in brackets): EcoRI (6‐FAM)‐ACA/MseI‐CTG, EcoRI (VIC)‐ACG/MseI‐CTA, and EcoRI (NED)‐ACC/MseI‐CTC. Purification and visualization of PCR products were performed as described in Rebernig et al. (2010). All samples were processed in a single PCR round. Fourteen samples (10%) were used as replicates to test reproducibility.

### Analysis of AFLP data

Peaks of relative fluorescent intensity were exported from AFLP electropherograms with GeneMarker version 1.8 (SoftGenetics, State College, PA) using default peak detection and then subjected to automated binning and scoring using RawGeno version 2.0 (Arrigo et al. 2009), a package for the software R (R Core Team, 2014). We applied the following settings: scoring range = 50–500 bp, minimum intensity = 100 rfu, minimum bin width = 1 bp, and maximum bin width = 1.5 bp. Fragments with a reproducibility in sample‐replicate comparisons lower than 80% were eliminated. The error rate (Bonin et al. 2004) was calculated as the ratio of mismatches (scoring of 0 vs. 1) over phenotypic comparisons in AFLP profiles of replicated individuals. Monomorphic fragments and those present/absent in all but one individual were removed from the dataset to avoid biased parameter estimates (Bonin et al. 2004).

Interspecific gene flow was inferred with NewHybrids version 1.1 beta (Anderson and Thompson 2002), which allows for the accommodation of dominant multilocus markers such as AFLPs (Anderson 2008). The posterior probability that each sampled individual belongs to each of several classes (parents, F1 and F2 hybrids, backcrosses) is computed by Markov chain Monte Carlo (MCMC) in a Bayesian model‐based clustering framework. The probability of class membership was computed without prior information on hybrid status, applying the “Uniform‐like priors” for parameter estimation and using 1.5 million MCMC generations following a burn‐in of 100,000 sweeps. We set an arbitrary threshold for parents (*K*. *arvensis*,* K*. *carinthiaca*) of ≥90% probability of membership in one of the two parental groups. Individuals with lower values were treated as hybrids in the downstream statistical analyses. To assess the power of RGS measurements in detecting individuals of presumed hybrid origin, hybrid individuals were further divided into one of four hybrid classes (F1, F2, backcrosses to either parent), according to the highest posterior probability.

In order to complement the Bayesian approach with distance‐based analyses, a principal coordinate analysis (PCoA) based on a Jaccard distance matrix of AFLP data was computed with Past version 2.17 (Hammer et al. 2001) and a Neighbor‐net was produced with SplitsTree version 4.13.1 (Huson and Bryant 2006), applying 1,000 replicates in bootstrap analysis.

### Analysis of ecological data

Environmental conditions were characterized by mean Landolt indicator values (Landolt 2010) of accompanying vascular plant species in herb and shrub layers calculated for circular plots centered at each target individual. Landolt indicator values describe ecological requirements of species in terms of climate (temperature, T; continentality, K; light, L) and soil parameters (moisture, F; reaction, R; nutrients, N; humus content, H; aeration, D; moisture variability, W) ranging from 1 (low) to 5 (high). Niche differences among the two parental species and their hybrids (identified with NewHybrids, see Results) were tested by comparing averaged indicator values of accompanying species in a multivariate analysis of variance (MANOVA) in R version 3.0.2. Visualization of particular ecological niches was performed using a principal component analysis (PCA) with Canoco version 5 (Microcomputer Power, Ithaca, US), based on standardized indicator values. Differences in additional environmental factors recorded at sites were inferred in R, either using an analysis of variance (ANOVA) followed by Tukey's HSD post hoc test (coverage of vegetation layers, species diversity) or, if the assumptions of ANOVA were not met, using a Kruskal–Wallis rank_ sum test followed by a multiple comparison test available in the package Pgirmess version 1.5.9 (Giraudoux 2014; bare rock cover).

### Analysis of morphological data

The morphological characters traditionally used for distinguishing *K*. *carinthiaca* from *K*.* arvensis* (Fischer et al. 2008) were included, except for flower color. Nine morphological characters were scored on sampled leaves: total length, maximum width, length and maximum width of the terminal lobe, length and maximum width of a middle lateral lobe, number of lateral lobes, degree of division (entire, lobed, pinnatifid, bipinnatifid), and number of hairs on the abaxial side along a 2‐mm‐long transect outside a main vein (mean value of three measurements). The set of morphological characters was supplemented with the ratio of the length of the terminal lobe and the total leaf length. The degree of phenotypic overlap among the parental taxa and their hybrids was inferred by a canonical discriminant analysis (CDA) conducted with Canoco. We tested the morphological discrimination among the three a priori defined groups (*K*. *arvensis*,* K*. *carinthiaca* and hybrids) based on the NewHybrids analysis of the AFLP data. Prior to the analysis, accessions with undivided leaves were excluded (due to missing data), and the dataset was checked for normal distribution of character values and for potential strong correlation of characters (i.e., with absolute values of correlation coefficients >0.95) using a set of R functions contained in MorphoTools version 1.01 (Koutecký 2015). In CDA, both marginal effect of particular characters and their conditional effects in a stepwise selection of the most informative characters were assessed using a Monte Carlo test with 9,999 permutations (Lepš and Šmilauer 2014). The corresponding significance levels were adjusted using Bonferroni correction (*P*‐value/number of characters in CDA).

## Results

### AFLPs

The three AFLP primer combinations yielded a total of 490 scored fragments of which 99 did not pass quality thresholds in RawGeno. Afterward, 83 fragments with singular presences or absences were manually excluded from the dataset. Of 143 sampled individuals, 15 failed to produce reliable AFLP profiles and were excluded. The final dataset thus consisted of 128 individuals and 308 AFLP fragments. Based on 14 replicates, the average error rate was 4.0%.

**Figure 2:**
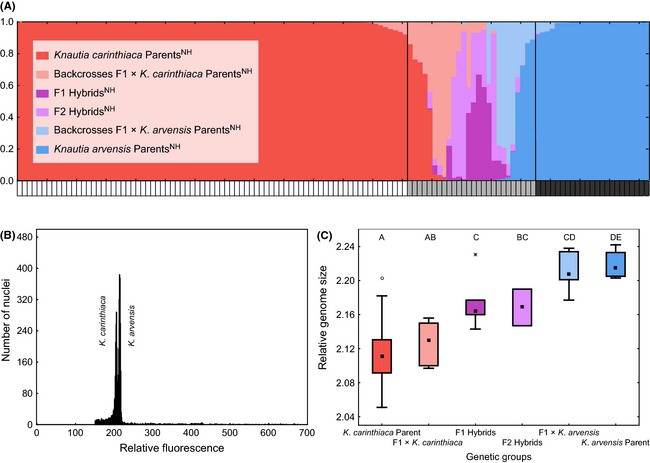
Molecular and cytological evidence of interspecific gene flow between *Knautia carinthiaca* and *K*. *arvensis*. (A) NewHybrids analysis with posterior distributions of two parental and four hybrid classes across the sampled individuals. The lower bar indicates whether particular individuals are treated as *K*. *carinthiaca* (white), *K*. *arvensis* (black), or hybrids (gray) in downstream analyses. Nine non‐admixed individuals of *K*. *arvensis* are from geographically distant reference populations collected outside Mt. Gutschenkogel. (B) Simultaneous flow‐cytometric analysis of a randomly selected individual from both parental species, illustrating a 4.3% difference in their relative fluorescence intensities. (C) Differences in relative genome size (square = median, box = upper and lower quartile, whiskers span the non‐outlier range) among individuals clustered into six genetic groups according to the NH analysis shown in (A). Different letters indicate significantly (*α* = 0.05) different groups in pair‐wise comparisons in Tukey's post hoc tests.

**Figure 3:**
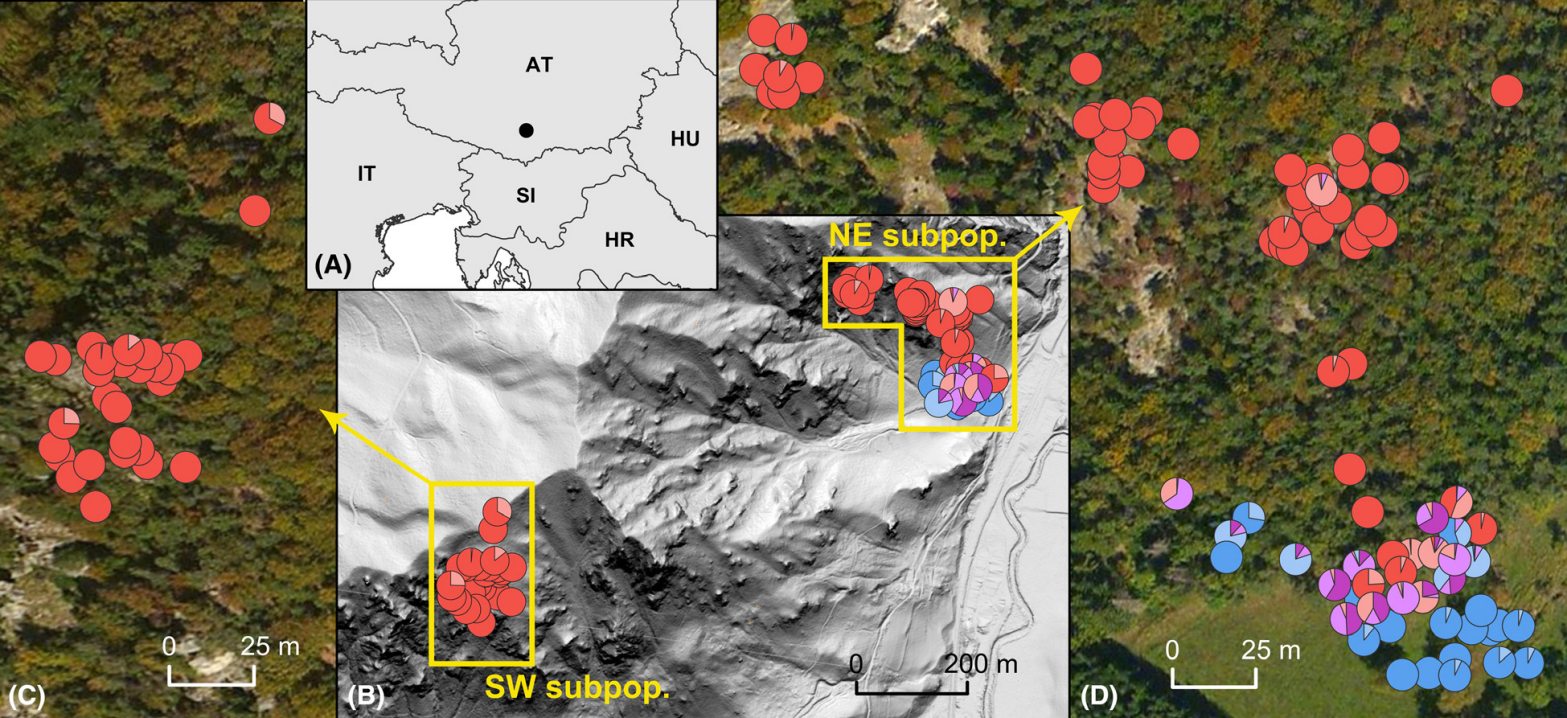
Spatial distribution of sampled *Knautia carinthiaca* individuals (red), covering the entire known distribution range of the species, adjacent *K*. *arvensis* individuals (dark blue), and their hybrids (other colors). Pie charts show hybrid classes based on NewHybrids analysis with the same color coding as in Fig. 2. (A) Location of study sites. (B) Distribution of the separated *K*. *carinthiaca* localities at Mt. Gutschenkogel plotted on a relief map; areas inhabited by *K*. *carinthiaca* are magnified in (C) and (D). (C) Detail of the forest‐bound SW locality with a minimal degree of introgressive hybridization from *K*. *arvensis*. (D) Detail of the NE locality neighboring a meadow with higher intensity of interspecific gene flow concentrated to the forest margin.

**Figure 4:**
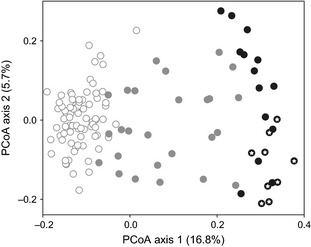
Principal coordinate analysis of Jaccard distances among AFLP multilocus phenotypes of *Knautia carinthiaca* (white), hybrid (gray), and *K*. *arvensis* (black) individuals. Samples from *K*. *arvensis* reference populations collected outside Mt. Gutschenkogel are marked with an asterisk.

**Figure 5:**
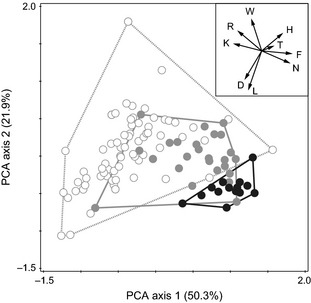
Principal component analysis reconstructing ecological niches of *Knautia carinthiaca* (white), *K*. *arvensis* (black), and their hybrids (gray) according to mean Landolt indicator values (LIV) of sites inhabited by the *Knautia* individuals at Mt. Gutschenkogel. Lines connect the most divergent individuals of a cluster.The inset shows relationships among the particular LIV values projected in the same ordination space as the samples: D, aeration; F, moisture; H, humus content; K, continentality; L, light; N, nutrients; R, reaction; T, temperature; W, moisture variability.

**Figure 6:**
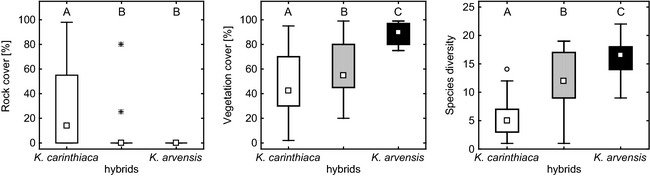
Ecological differences among *Knautia carinthiaca* (white), *K*. *arvensis* (black), and their hybrids (gray) represented by three additional significant environmental variables recorded in situ at Mt. Gutschenkogel (square = median, box = upper and lower quartile, whiskers span the non‐outlier range). Different letters indicate significantly (*α* = 0.5) different groups in pair‐wise comparisons in Tukey's post hoc tests.

**Figure 7:**
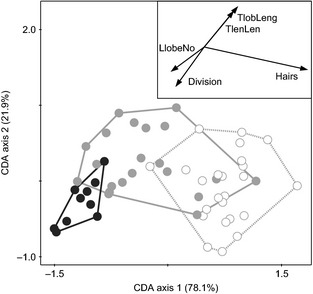
Canonical discriminant analysis of *Knautia carinthiaca* (white, *N* = 24), hybrid (gray, *N* = 21) and *K*. *arvensis* (black, *N* = 10) individuals sampled at Mt. Gutschenkogel based on ten morphological characters. Lines connect the most divergent individuals of a cluster. Arrows in the inset represent relationships of five characters with significant independent contributions to the overall explained variation (i.e., marginal effects): Hairs, hair density; TlobLeng, length of the terminal lobe; Division, degree of leaf division; LlobeNo, number of lateral lobes; TlenLen, ratio length of the terminal lobe/leaf length.

NewHybrids identified 25 hybrid individuals using an arbitrary posterior distribution threshold (<90% probability of membership in one of the two parental groups; Fig. 2), and this categorization formed the basis for the downstream analyses depicted in Figs. 3, 4, 5, 6, 7. Hybrid individuals were further divided into four classes: F1 Hybrids^NH^ (five individuals), F2 Hybrids^NH^ (four individuals), F1 × *K*. *arvensis* ^NH^ (eight individuals), and F1 × *K*. *carinthiaca* ^NH^ (nine individuals). Individuals identified as pure parental species (*K*. *carinthiaca* Parent^NH^, *K*. *arvensis* Parent^NH^) formed two clearly separated groups in a Neighbor‐net with strong bootstrap support (92.6%; data not shown). Most hybrid individuals, spanning all four hybrid classes, were from the NE locality, where *K*. *arvensis* occurs in close proximity to *K. carinthiaca* on an adjacent pasture. The only sign of interspecific gene flow in the SW locality were three individuals classified as F1 × *K*. *carinthiaca* ^NH^ hybrids (Fig. 3). Principal coordinate analysis (PCoA) of AFLP data resulted in clear separation of *K*. *arvensis* and *K*. *carinthiaca* individuals along the first axis, with hybrids filling the gap between their parents (Fig. 4).

### Relative genome size

Flow‐cytometric screening revealed only tetraploid individuals on Mt. Otwinskogel likely belonging to *K. norica* (see Discussion), thus showing that diploid *K. carinthiaca* is restricted to a single population on Mt. Gutschenkogel. At Mt. Gutschenkogel, we found altogether 117 diploid individuals in both subpopulations and a single triploid individual in the NE locality; the triploid was retained in all analyses except for the RGS comparisons. On the diploid level, differences in the RGS between individuals determined by AFLP genotyping as *K*. *carinthiaca* Parent^NH^ (2.11 ± 0.03) and *K*. *arvensis* Parent^NH^ (2.22 ± 0.02) were significant (*F*_1, 76_ = 188.5, *P* < 0.001). Flow‐cytometric analysis of a mixed sample containing leaf tissue of both species resulted in a histogram with two peaks, confirming that the approximately 4.3% difference in relative fluorescence intensities is not an instrumental artifact (Fig. 2B). The RGS of individuals with hybrid status as identified by the NewHybrids analysis of AFLP data (2.17 ± 0.04) was significantly different from RGS values of both parental taxa (ANOVA followed by Tukey's post hoc test; F_2, 98_ = 82.4, *P* < 0.001). Further division of hybrid individuals into four hybrid classes (based on NewHybrids analysis of AFLP data) found also some, albeit limited, support in RGS measurements (Fig. 2C).

### Ecological niches

Significant differences in ecological niches among the two parental taxa and their hybrids were confirmed using multivariate analysis of variance (MANOVA: *F*_18, 210_ = 12.6, *P* < 0.001). In a principal component analysis (PCA) diagram, *K*. *arvensis* and *K*. *carinthiaca* individuals were separated (Fig. 5), suggesting that parental niches are distinct. Several LIVs corresponded with the distinction of the two parental niches in PCA: *K. arvensis* inhabited moister (F) and more nutrient‐rich (N) sites, whereas *K*. *carinthiaca* preferred sites with higher pH (R), more intense moisture variability (W), and higher proportion of accompanying species with continental distribution tendencies in Europe (K). Hybrid individuals had intermediate ecology in most cases; a few fell into the niches of one of their parents.

Significant differences were also found in additional ecological variables characterizing the niches (Fig. 6): rock cover (*Χ* ^2^ = 21.99, df = 2, *P* < 0.001), herb cover (*F*_2, 110_ = 23.01, *P* < 0.001), and accompanying species diversity (*F*_2, 110_ = 71.23, *P* < 0.001), but not moss cover (*Χ* ^2^ = 5.97, df = 2, *P* = 0.0504). Rock cover and herb cover were negatively correlated (Spearman′s rho = −0.63). Specifically, *K*.* carinthiaca* differed from both hybrids and *K*. *arvensis* in frequent occurrence in rock crevices. On the other hand, *K*. *arvensis* inhabited sites with more dense vegetation and higher species diversity as compared to *K*. *carinthiaca*; hybrid individuals mostly grew at intermediate sites.

### Morphology

Data on morphology were available for 24, 10, and 21 individuals of *K*. *carinthiaca*,* K*. *arvensis,* and hybrids, respectively. Most characters were normally distributed, only two of them (degree of leaf division, number of lateral lobes) deviated from normality. No highly correlated character pairs (i.e., with absolute values of correlation coefficients >0.95) were found using either Pearson or Spearman correlation coefficient. Canonical discriminant analysis (CDA) based on nine measured and one derived morphological character separated well the two parental species (Fig. 7, *P* < 0.001 under 9,999 permutations). Hybrid individuals were either of intermediate morphology or resembled one of their parents. Five morphological characters explained a significant part of the overall variation when used as the only predictors in independent tests (i.e., marginal effect, Table 1). However, only two characters showed significant unique contribution when included into the model during a stepwise selection of best model predictors (i.e., conditional effect, Table 1). Application of Bonferroni correction reduced the number of morphological characters with significant marginal and conditional effects to four and one, respectively. The most informative character was hair density, explaining 65.3% of the overall variation.

**Table 1:** List of morphological characters with a significant independent contribution to the overall explained variation (i.e., marginal effect) based on Monte Carlo test in CDA. Additionally, unique contributions of these characters (i.e., their conditional effects) were also tested during a stepwise selection of best CDA predictors. *P*‐values significantly different (*α* = 0.5) after applying Bonferroni correction are marked with an asterisk

Morphological character	Abbrev. in CDA	Independent contribution (marginal effect)			Unique contribution (conditional effect)		
		Contribution	*F*	*P*	Contribution	*F*	*P*
Hair density	Hairs	72.7%	26.9	<0.001*	72.7%	26.9	<0.001*
Length of the terminal lobe	TlobLen	24.2%	6.7	0.001*	11.7%	4.6	0.019
Degree of leaf division	Division	19.8%	5.4	0.005*	n.s.	n.s.	n.s.
Number of lateral lobes	LlobeNo	19.7%	5.3	0.005*	n.s.	n.s.	n.s.
Ratio length of the terminal lobe/leaf length	TlenLen	19.6%	5.3	0.006	n.s.	n.s.	n.s.

## Discussion

### The occurrence of *Knautia carinthiaca* is limited to a single population

*Knautia carinthiaca,* a local endemic of the northern Klagenfurter Becken in Kärnten/Carinthia, southern Austria (Schratt‐Ehrendorfer 2009), was described relatively recently as a new species based on its specific morphology and ecological requirements, as well as on the wide disjunction separating it from presumed close relatives on the Balkan Peninsula (Ehrendorfer 1962). It is likely a relict species that underwent a major bottleneck during the Holocene, when forest expansion caused a massive decline of populations of many light‐demanding plant species in Central Europe (Birks and Willis 2008), including other representatives of *Knautia* (Kolář et al. 2012). Typical refugia for light‐demanding plant species include the core habitats of *K*. *carinthiaca*, that is, limestone and dolomite outcrops and adjacent open forests and dry grasslands, where establishment of competitors is constrained by edaphic factors (Pigott and Walters 1954; Kaplan 2012).

Our molecular‐genetic, flow‐cytometric, and morphometric data are clearly consistent with an independent evolutionary status of *K*. *carinthiaca*, supporting the conclusions of Rešetnik et al. (2014), which were based on a limited number of *K. carinthiaca* samples. Individuals identified as *K*. *carinthiaca* Parent^NH^ and *K*. *arvensis* Parent^NH^ by NewHybrids analysis (Fig. 2A) formed two clearly separated groups also in other analyses of AFLP data (Fig. 4), showing marked genetic differentiation of these two sympatric species. The divergence of *K*. *carinthiaca* was also apparent when *K*. *norica* and *K*. *drymeia*, two closely related sympatric tetraploid species, were included in AFLP analyses (M. Čertner, F. Kolář, P. Schönswetter and B. Frajman, unpubl. data). In the same line, relative genome size (RGS) consistently differed by 4% between *K*. *carinthiaca* and *K*. *arvensis*; simultaneous flow‐cytometric analyses of both species always resulted in histograms with two peaks (Fig. 2B), supporting that the observed difference is no instrumental error (Greilhuber 2005). These findings further support the potential of flow cytometry as a suitable nondestructive and high‐throughput tool for fast assessment of hybridization risks in plant conservation (see also Hanušová et al. 2014; Kabátová et al. 2014; Vít et al. 2014), which is based on the observation that nuclear DNA content may differ substantially among homoploid species of the same genus (Bennett and Leitch 2011), but is mostly stable within a species (Greilhuber 2005).

The distribution of *K. carinthiaca* was suggested to span two mountains: the locus classicus Mt. Gutschenkogel west of Eberstein in Görtschitztal and Mt. Otwinskogel north of Launsdorf (Ehrendorfer 1962; Melzer 1973; Schratt‐Ehrendorfer 2009). It is fairly unlikely that *K. carinthiaca* occurs at other localities in the region, as the distribution of vascular plants in Carinthia is well documented (Hartl et al. 1992). Flow‐cytometric screening of 118 and 50 individuals, respectively, collected at both localities revealed that the species is actually restricted to its locus classicus. Its distribution might be slightly larger than indicated in Fig. 3, possibly including inaccessible rock faces not sampled by us. On the second locality, Mt. Otwinskogel, no diploid individuals were found, and only tetraploid *K*. *norica* inhabited the site. Apart from ploidy level, *K*. *carinthiaca* and its presumed allotetraploid‐descendent *K*. *norica* (Ehrendorfer 1962) differ in monoploid genome size and form well‐separated clusters in AFLP analyses (M. Čertner et al., unpubl. data). This rejects the hypothesis that plants on Mt. Otwinskogel are autotetraploid derivatives of *K*. *carinthiaca*. Evidently, we can neither exclude that *K*. *carinthiaca* occurred on Mt. Otwinskogel and was recently drawn to extinction, nor, alternatively, that we have overlooked the species. We strongly believe, however, that the record was based on misidentification of *K*. *norica* for four reasons. First, *K*. *carinthiaca*, similarly as *K*. *arvensis* ssp. *serpentinicola* (Hanzl et al., 2014), is a long‐lived perennial and the first record dates back only ca. 50 years; it is thus highly improbable that the entire population went extinct in the meantime. Second, we searched for *K*. *carinthiaca* in 2010 when collecting samples for the study by Rešetnik et al. (2014) and, additionally, in 2013 and visited all accessible habitats. Third, on Mt. Otwinskogel, *K*. *norica* inhabits habitats such as crevices in vertical rock cliffs that are typical for *K. carinthiaca* and some individuals morphologically strongly resemble *K. carinthiaca* (authors' personal observations). Fourth, we could not trace reliably determined herbarium vouchers from Mt. Otwinskogel.

### Introgressive hybridization with a widespread congener

Both distance‐ and model‐based analyses of AFLP data support the occurrence of interspecific hybrids between *K*. *arvensis* and *K*. *carinthiaca*, which is in accordance with significant differences in RGS between AFLP‐based hybrid and parental groups and corroborates previous field observations (Schratt‐Ehrendorfer 2009). NewHybrids classified AFLP profiles of the sampled individuals into two parental and four hybrid classes (Fig. 2A). Apart from nine presumable F1 and F2 hybrid individuals, two‐thirds of the hybrids showed signs of backcrossing with the parental species. Hybrid classes are paralleled by gradual changes in RGS (Fig. 2C), lending strong support to the hypothesis that introgressive hybridization between *K*. *carinthiaca* and *K*. *arvensis* results in the formation of vital and fertile hybrids, as frequently observed in other homoploid crosses between *Knautia* species (Ehrendorfer 1962; Kolář et al. 2012; Rešetnik et al. 2014). There is no evidence for a bias with respect to backcrosses with one or the other parent as similar numbers (eight and nine, respectively) of individuals were classified as F1 × *K*. *arvensis* ^NH^ and F1 × *K*. *carinthiaca* ^NH^. Such symmetry in frequency of backcrossing was also reported from other hybridizing plant and animal groups (Travnichek et al. 1997; Minder et al. 2007; Raudnitschka et al. 2007; Hanušová et al. 2014).

*Knautia carinthiaca* and *K*. *arvensis* are morphologically clearly distinct (Fig. 1). Morphological characters with significant independent contributions in the canonical discriminant analysis (Fig. 7; Table 1) included three traditionally used characters, that is, hair density, number of lateral leaf lobes, and length of the terminal leaf segment. Hair density contributed 65% of the overall phenotypic diversity, which is in accordance with our field observations that *K*. *carinthiaca* differs from *K*. *arvensis* mostly in having dense, soft indumentum. Interspecific hybrids either displayed intermediate character states or were similar to either parent (Fig. 7). Continuous variability of hybrid phenotypes ranging from *K*. *carinthiaca*‐like to *K*. *arvensis*‐like morphology is in accordance with the wide variety of hybrid classes including backcrosses with either parent. Morphological investigations are thus insufficient for the assessment of hybridization risk in *K. carinthiaca* as the phenotypic similarity of the backcrosses to one of the parents could lead to serious underestimation of the levels of introgression between the species.

### Environmental barriers to gene flow

The ecological niches of *K*. *carinthiaca* and *K*. *arvensis* differ substantially. While the former favors rocky sites with sparse and species‐poor vegetation, *K*. *arvensis* is associated with dense, species‐rich vegetation (Fig. 5). This is in agreement with our field observations that *K*. *carinthiaca* mostly occupies crevices of limestone or dolomitic rocks as well as adjacent open forests or dry grasslands with poorly developed soils, whereas *K*. *arvensis* is confined to moist and nutrient‐rich sites such as meadows, pastures, roadsides, and other anthropogenic grasslands. Numeric comparison of ecological niches of both species revealed that there is almost no overlap between the core niches of *K*. *carinthiaca* and *K*. *arvensis*, but they can come into contact in marginal habitats. We observed such contact at a forest margin (lowest part of the NE locality, Fig. 3), where scattered *K*. *carinthiaca* individuals neighbor an extensively managed grassland inhabited by *K*. *arvensis*. Most hybrids (85% of 26) spanning all hybrid classes were collected at this particular site, either on the pasture or in the forest but only rarely >20 m from the forest margin, which obviously represents an ecological transition between the parental core niches (Fig. 3). Conversely, only four hybrid individuals (15%; invariably F1 × *K*. *carinthiaca* ^NH^) were identified among plants from limestone outcrops in the core of the *K. carinthiaca* population (Fig. 3).

Contrasting ecological preferences of *K*. *carinthiaca* and *K*. *arvensis* on the one hand and the forested area surrounding the core of the *K. carinthiaca* population on the other hand likely act as efficient barriers to interspecific gene flow and prevent introgression from *K. arvensis* into *K. carinthiaca*. This environmental barrier is, however, permeable in peripheral habitats such as forest margins, where the two species coexist and form hybrid swarms. Still, *K*. *carinthiaca* individuals separated from the closest *K*. *arvensis* population by >50 m of continuous forest were only exceptionally introgressed. Given that both species are insect pollinated and differ slightly in flower color (Schratt‐Ehrendorfer 2009) but not in floral shape, which is highly conserved throughout *K*. sect. *Trichera* (Rešetnik et al. 2014), we hypothesize that habitat segregation is the preeminent prezygotic barrier to pollination. Moreover, even if the fruits of *Knautia* are distributed by ants due to their lipid‐rich elaiosome (Mayer and Svoma 1998; Schratt‐Ehrendorfer 2009), it appears more likely that due to gravity, fruits of *K. carinthiaca* are spontaneously transferred downhill toward the population of *K. arvensis* than vice versa. In addition, postzygotic barriers might be involved in reducing hybridization between *K*. *arvensis* and *K. carinthiaca*. Whereas hybrids are likely fertile (see above), hybrid progeny might be maladapted to the parental core niches, prospering only in intermediate habitats (Stebbins 1950; Arnold 2004). This hypothesis would require further study by reciprocal transplant experiments; however, such approach is not conceivable as it presents a strong conservational risk for *K*. *carinthiaca*.

### Conservation of *K. carinthiaca*

Endemic species, especially those restricted to a single locality or population, are top‐ranking priorities of both national and worldwide conservational efforts (Myers et al. 2000; Cook and MacDonald 2001; Brooks et al. 2002). *Knautia carinthiaca* is listed in the Red List of Pteridophytes and Spermatophytes of Carinthia (Kniely et al. 1995) and the Red List of endangered plants of Austria (Niklfeld and Schratt‐Ehrendorfer 1999) as potentially vulnerable. We propose the category Critically Endangered (CR) for *K*. *carinthiaca* as the species meets the following IUCN (2012) criteria: species known to exist at only a single location, area of occupancy <10 km^2^, and continuing decline in extent of its habitat was observed (expansion of a nearby quarry, see below).

Introgressive hybridization between *K*. *carinthiaca* and *K*.* arvensis* takes place mostly at the forest margin (Fig. 3); plants occupying core niche habitats were only exceptionally introgressed. The >50 m wide forest belt separating the pasture from the core habitats of *K*. *carinthiaca* seems to effectively prevent interspecific hybridization and thus reduces the extinction risk via genetic erosion. Moreover, sparse and isolated occurrence of *K*. *carinthiaca* in this forest belt likely reflects only local presence of suitable microhabitats and hence constrains further spread of introgressed hybrids toward the core habitats. At present, *K*. *carinthiaca* is thus relatively well sheltered from genetic erosion. However, further expansion of the nearby quarry into the vicinity of the *K. carinthiaca* population (which already took place between 2010 and 2013) and ruderalisation of the surrounding area could provide new suitable habitats for *K. arvensis* in close vicinity of *K. carinthiaca*. Therefore, as any major change in forest use (clear cutting, ruderalisation) strongly increases the risk of immigration of and introgressive hybridization with *K*. *arvensis*, a total ban of both deforestation and the expansion of the nearby quarry seems desirable, which might be easiest accomplished by establishing a nature conservation area including the rocky habitats inhabited by *K. carinthiaca* plus an at least 100 m broad forest buffer area.

## Data Accessibility

The datasets used in the study (AFLP binary matrix, genome size data, ecological characterization of habitats, morphological measurements) are available in Dryad repository; doi: 10.5061/dryad.6dn80.

## Conflict of Interest

None declared.
